# Early extracorporeal membranous oxygenation and burn excision in severe burn and inhalation injury

**DOI:** 10.1177/20595131241302942

**Published:** 2024-12-11

**Authors:** Andrew P. Bain, Isabel Garcia, Matthew Leveno, Chiaka Akarichi

**Affiliations:** 1 Department of Surgery, 12334 University of Texas Southwestern Medical Center, Dallas, TX, USA; 2 Department of Internal Medicine, University of Texas Southwestern Medical Center, Dallas, TX, USA

**Keywords:** ECMO, ECLS, burn excision, ARDS, burn

## Abstract

**Introduction:**

Extracorporeal membranous oxygenation (ECMO) as a salvage therapy for patients with severe acute respiratory distress syndrome (ARDS) has been described but experience is limited in burn cases. Few case reports detail the use of ECMO the setting of burn excision.

**Case:**

Here, we describe a 40-year-old female found down in a house fire who presented with 30% total body surface area burns and severe inhalation injury resulting in ARDS. Veno-venous ECMO was initiated 12 h after injury, with a total ECMO run of 523 h. In that time, she underwent three tangential excisions with significant intraoperative and postoperative bleeding complications requiring in total 37 units of packed red blood cells, 8 pools of platelets, 24 units of fresh frozen plasma, and 1 unit of cryoprecipitate. The patient was successfully weaned from veno-venous ECMO. She required six subsequent excisions after her ECMO decannulation for both infection control and complete excision of her full-thickness burns. She was ultimately discharged to an inpatient rehabilitation facility.

**Discussion:**

This report serves as the first detailed description of perioperative resuscitation on ECMO during burn excision and adds to the body of literature regarding ECMO support in the burned patient. This case specifically highlights the multidisciplinary care and resource demands of performing burn excision during ECMO as well as the associated bleeding complications of doing so. Further study is needed to define optimal timing, patient selection, and strategy for coagulopathy management and surgical care of the burn patient with ARDS treated with ECMO.

**Lay Summary:**

Patients with severe burn injuries can have associated injuries to their lungs from both smoke and as a response to the stress a severe burn puts on the body. The injuries can be so severe that supportive machines can be needed that do the work of the lungs by adding oxygen to the blood, called extracorporeal membranous oxygenation (ECMO). These extreme measures are critical to supporting severe respiratory problems and have been incorporated into caring for burn patients with severely injured lungs. ECMO requires significant resources and has risks, including bleeding and clotting issues. Severely burned patients also need surgery to remove burned skin and decrease the stress placed on the body. Only a handful of cases have been described where burn surgery has been performed while a patient was on ECMO support. In our experience caring for a severely burned patient and performing multiple surgeries on ECMO, we encountered multiple bleeding complications secondary to the use of ECMO, resulting in large amounts of transfusion products needed. After one month, the patient's lungs recovered and ECMO was not needed. The patient survived to discharge from the hospital after completion of additional necessary burn surgeries. This report is the first to explain in detail the experience of performing burn excision on a patient on ECMO. We describe the resources and team members needed for success and believe further research must be done to best manage burn injuries while on ECMO.

## Introduction

In the intubated burned patient, cute respiratory distress syndrome (ARDS) is common, with incidence rates ranging from 30%-40%.^1,2^ The mechanism of lung injury in severe burns is multifactorial. Systemic vascular hyperpermeability from cutaneous burns leads to interstitial edema in the lungs.^3^ Increased bronchial blood flow secondary to direct inhalational injury results in edema, increased mucus secretion and plasma transudation, and the formation of casts from epithelial debris, fibrin clots, and mucus.^4^ ARDS in the burned patient carries significant mortality, estimated at 31%.^5^

The use of extracorporeal membranous oxygenation (ECMO) in patients with severe neonatal and pediatric respiratory failure is well-established.^6^ In the adult population, ECMO as a salvage maneuver in severe ARDS has been well described but routine use is seen as controversial, lacking strong supporting evidence from robust randomized trials. However, the CESAR trial in 2009 described reduced mortality in patients with severe respiratory failure due to a reversible cause who were referred to an ECMO center.^7^ In the burn population, the use of ECMO as a salvage therapy for severe ARDS is limited to case reports and case series. Here, we present a case of early ECMO utilization in the management of severe ARDS secondary to both inhalational and burn injury.

## Case presentation

A 40-year-old female with an unknown medical history and Class III obesity (BMI 50.2) presented after being found down, prone and unconscious, in a house fire. The patient was estimated to have been in the house for at least 15 min. She was unresponsive and intubated on scene. After arrival to our burn facility, the patient had 30% total body surface area (TBSA) full-thickness burns to the head, posterior neck, chest, abdomen, bilateral upper extremities, and bilateral lower extremities. Escharotomies were performed to the right upper extremity and hand due to circumferential full-thickness burns with an undetectable radial pulse. There was improvement in her radial artery Doppler signal after escharotomy. On arrival, her neurologic status included movement of all extremities spontaneously prior to sedation and paralysis, but she did not follow commands. Additionally, the patient was severely hypoxic and hypercapnic, with Sa02 in the range of 30–40% and PaCO2 at 114 mmHg (15.1 kPa). Due to difficulty in oxygenation, multiple attempts were made to rescue oxygenation with manual bag valve ventilation on FiO2 100%. Despite manual ventilation attempts the patient remained severely hypoxic. Multiple ventilation modes were attempted without improvement in oxygenation or ventilation. She ultimately was placed on pressure control ventilation, with PIP ranging 46–48 mmHg (6.1 kPa), maximum PEEP 20 mmHg (2.7 kPa), rate of 38 breaths/minute, FiO2 100%. Achieved tidal volumes continued to decrease, from 537cc to 240cc. Chest X-ray demonstrated bilateral infiltrates. Pink frothy secretions were noted in the endotracheal tube, and bronchoscopy was performed with evidence of carbonaceous sputum in the lower airways. Despite maximum vent settings, paralysis, and the addition of nitric oxide at 40 ppm, she continued to deteriorate and was transferred to the intensive care unit (ICU). Given her persistent severe hypoxemic respiratory failure, evidence of severe inhalation injury, and the expected worsening of her pulmonary function during the remaining large volume burn resuscitation, the ECMO team was consulted who agreed with her need for emergent total pulmonary support. Twelve hours after injury, veno-venous (VV) ECMO was initiated via bilateral femoral vein cannulation. After ECMO cannulation, her family was finally able to be contacted, and we learned of the patient's history of developmental delay. While she lived with family, she was independent in her activities of daily living and functioned approximately at the level of a 12-year-old. The patient remained on ECMO for a total of 22 days, approximately 523 h.

The patient underwent multiple debridements of her burn wounds while on ECMO. On hospital day 6, tangential excision of the right upper extremity and left lower extremity were performed with 8% TBSA of full-thickness burns excised and covered with allograft resulting in 700 cc of intraoperative blood loss. Hemostasis was achieved with the application of thrombin/epinephrine solution to the wound bed and hemostatic dressings. The patient required 2 units of packed red blood cells (pRBCs) and 1 pool of platelets intraoperatively. Postoperatively, due to bleeding from excisions, she required another 2 units of pRBCs, 1 pool of platelets, and hemostasis at bedside in the ICU with electrocautery, hemostatic suturing, and pressure. Therapeutic heparin, used to prevent clotting in the ECMO circuit, was stopped 6 h prior to operation and restarted on postoperative day 0, 10 h after her return to the ICU after hemostasis was assured at excision sites. On hospital day 9, heparin was adjusted to subtherapeutic dosing due to bleeding from excision sites. On hospital day 10, heparin was stopped 6 h prior to the operation, and the patient was taken for tangential excision with electrocautery of 12% TBSA full-thickness burns down to the level of the subcutaneous tissue on the chest and abdomen, followed by placement of an allograft, with 1000 cc of intraoperative blood loss. Again, surgery was complicated by significant intraoperative bleeding, requiring 4 units of pRBCs, 4 units of fresh frozen plasma (FFP), and 1 pool of platelets. Upon return to the ICU, there were continued issues with achieving adequate ECMO flows due to hypovolemia caused by continued coagulopathic bleeding. Surgical dressings were taken down and electrocautery, hemostatic suturing, pressure, and topical hemostatic agents with thrombin/epinephrine-soaked gauze were employed. Massive transfusion protocol was initiated, and the patient received 16 units pRBCs, 10 units of FFP, 2 pools of platelets, and 1 unit of cryoprecipitate. Hemostasis was achieved through both blood product resuscitation and bedside surgical management. Anticoagulation was not restarted given continued bleeding from excisional sites. The remainder of the ECMO run occurred without anticoagulation and without any significant clotting events. On hospital day 17, primary excision of the right thigh and repeat excision of the right upper extremity, right hand, and chest for control of fungal infections were completed with 2000 cc of intraoperative blood loss. Massive transfusion was required intraoperatively due to hemorrhage during excision. The patient received 11 units of pRBCs, 1 pool of platelets, and 10 units of FFP intraoperatively. With improved intraoperative blood product resuscitation, she did not require active hemorrhage control in the ICU, and only minor oozing from excision sites was noted. The patient did have flow and suction events that evening, which were managed with albumin, 2 units of pRBCs, and 2 pools of platelets.

During the first three weeks of the patient's hospitalization, her respiratory status slowly improved. On day 4 of the ECMO run, her tidal volumes were 20 cc on pressure control ventilation with an inspiratory pressure of 18 mm Hg with a PEEP of 10 mm Hg, FiO2 of 100%, PIP of 28, PCO2 of 43, PO2 of 51, and P:F ratio of 51 with maximal VV ECMO support with a sweep of 14 and FiO2 of 100%. On day 20 of ECMO, the patient was successfully weaned from VV ECMO support, and she maintained adequate respiratory function on pressure control ventilation of 16/12 with an FiO2 of 40%, achieving a PCO2 of 49, PO2 of 113, and P:F ratio of 282. The patient underwent successful bedside percutaneous tracheostomy prior to decannulation of ECMO. The patient continued to tolerate a capped ECMO circuit, and she was successfully decannulated on day 22.

The patient's course was complicated by Klebsiella and Citrobacter ventilator-associated pneumonia. She also suffered pseudomonas bacteremia secondary to infected wounds and cutaneous mucormycosis of her wounds. She ultimately required six subsequent excisions after her ECMO decannulation for both infection control and complete excision of her full-thickness burns. On hospital day 60, she underwent final excision with split-thickness skin grafting of the bilateral upper extremities, bilateral lower extremities, chest, and abdomen. She passed a tracheostomy capping trial. Her tracheostomy was decannulated prior to discharge to an inpatient rehab facility on hospital day 95. She was ultimately discharged home and receives current care closer to her rural home.

## Discussion

### ECMO in the burned patient

ARDS is common among critically ill burn patients. The extent of full-thickness burns correlates with the incidence of moderate to severe ARDS and impacts overall mortality.^2^ ECMO has been used as a salvage therapy in patients with severe ARDS, but experience remains limited in the burn population.^7^ The first reported case of ECMO in a burn patient was described by Ombrellaro et al.,^8^ who successfully treated an 11-month-old boy with 32% TBSA burn and concomitant inhalation injury leading to ARDS in 1994. In 1996, Patton et al.^9^ described the first successful use of ECMO as salvage therapy in an adult with 12% TBSA cutaneous burn and bronchoscopically demonstrable inhalation injury and subsequent ARDS. Since then, subsequent case reports and series of institutional experience using ECMO as salvage therapy in burn-associated acute lung injury have emerged with varying degrees of morbidity and mortality reported. In 2013, Asmussen et al.^10^ performed a systematic review of the reported cases of ECMO in burn and smoke inhalation injury. Sixteen individual case reports were excluded from the review. In case series and retrospective reviews, the authors identified 68 patients as being treated with ECMO in the setting of burn injury, with a survival rate of 54%. The authors found a higher rate of survival in those with ECMO run times of less than 200 h compared with run times of 200 h or more.^10^ The run time for the case presented here is 523 h. Survival rates in the small case series reported since 2013 range from 16.7% to 80%.^11–15^ In a review of the Extracorporeal Life Support Organization registry, Burke et al.^13^ noted no survival difference between veno-arterial and VV ECMO. Both Burke et al.^13^ and Nosanov et al.^16^ determined that survival rates were similar between burn patients and nonburn patients receiving ECMO therapy, suggesting ECMO as a viable rescue option in burn patients. Of note, most reports focus on patients with inhalation injury and no operative needs or those needing ECMO after operative needs were addressed.

### Excision on ECMO

ECMO in the burned patient may better be understood in two groups, the first being isolated inhalation injury and second as those with burns requiring operative intervention. ECMO appears to be a viable option in burn-related ARDS. However, in the operative group, associated bleeding complications, as experienced in our case, must be considered, and may require significant resources and support. Reports on burn excision prior to or on ECMO remain limited. Bleeding is a common complication of ECMO, occurring in 13%-26% of all adult patients^17,18^ and remains an important consideration in the burn patient. ROTEM was used to aid our perioperative resuscitation. Holzer et al. correlated postoperative bleeding with fibrinogen levels in the unburned surgical patient on ECMO.^19^ Additionally, systemic anticoagulation is often used to avoid clotting issues in the ECMO circuit and can present challenges in the postoperative period. However, a 2021 systematic review suggests comparable bleeding and thrombosis events between anticoagulated and non-anticoagulated patients.^20^

Eldredge et al. reported eight cases of burn injury treated with ECMO, and five were noted to have bleeding complications. Three of the latter patients had burn excision prior to ECMO initiation, and in all three bleeding was noted from their excision sites.^21^ Szentgyorgyi et al.^12^ described one experience of debridement of a patient on ECMO, who required significant blood transfusion postoperatively. Ainsworth et al.^14^ reported a series of 14 burn patients treated with extracorporeal life support, six of whom underwent burn excision while on ECMO. Hsu et al.^11^ presented a series of six patients treated with ECMO for significant burns secondary to a stun grenade. Four patients were treated with veno-arterial ECMO and two with VV ECMO. These patients had a TBSA of 50%-99%, rhabdomyolysis, renal failure, and coagulopathy, and two patients had concomitant pneumothorax. All patients underwent debridement at the bedside while on ECMO with only one patient surviving to discharge. Details of these operations were not included.

Our patient underwent three excisions while on ECMO. These operations required tremendous coordination between the burn surgery team, ECMO specialists, cardiac anesthesiologists, transfusion providers, ICU staff, and operating room staff. Not only does the patient require safe transport to and from the operating room, the ECMO circuit requires constant vigilance. Coagulopathy secondary to shock and the ECMO circuit requires advanced planning to have necessary blood products on hand and an adept anesthesiology team to keep the patient adequately resuscitated. The excisions must be focused and expedient to limit operating time. Removing full-thickness eschar resulted in some physiologic improvement after each excision.

### Ethical considerations

ECMO is a resource intensive invasive intervention typically reserved for rescue therapy in the severely ill patient. A recent metanalysis reports the majority of burned patients receiving ECMO have ARDS and are undergoing VV ECMO, similar to our patient.^22^ ECMO is typically viewed as a bridge therapy, providing time for a patient's organ injury to recover or proceed to transplant.^23^ Degree of inhalation injury, degree of burn injury, and patient comorbidity should be considered in terms of potential recovery. In the emergent setting, access to a patient's medical history may be limited as in our case. In that situation, working from the highest level of cognitive function observed on physical exams or reports from emergency medical services may be the only information available. During the course of treatment, communication with the patient's surrogate decision maker is critical.^24^ During the most intense periods of illness for our patient, severity was communicated and decisions to persist in support with ECMO and other critical care modalities were respected, with an ultimate positive outcome in survival with good quality of life.

## Conclusion

In the severely burned patient, especially those with concomitant lung injury, ECMO can serve as a viable salvage option in severe ARDS. Our case of successful early initiation of ECMO and repeated tangential excision during ECMO with survival to discharge adds to our limited knowledge about extracorporeal life support in the burn population. This case specifically highlights the demands of performing burn excision during ECMO as well as the associated bleeding complications of doing so. Further study is needed to define optimal timing, patient selection, and strategy for coagulopathy management and surgical care of the burn patient with ARDS treated with ECMO.

## References

[1] Ranieri VM Rubenfeld GD Thompson BT , et al. Acute respiratory distress syndrome: the Berlin definition. JAMA 2012; 307: 2526–2533. 22797452 10.1001/jama.2012.5669

[2] Cartotto R Li Z Hanna S , et al. The acute respiratory distress syndrome (ARDS) in mechanically ventilated burn patients: an analysis of risk factors, clinical features, and outcomes using the Berlin ARDS definition. Burns 2016; 42: 1423–1432. 27520712 10.1016/j.burns.2016.01.031

[3] Enkhbaatar P Traber DL . Pathophysiology of acute lung injury in combined burn and smoke inhalation injury. Clin Sci (Lond) 2004; 107: 137–143. 15151496 10.1042/CS20040135

[4] Foncerrada G Culnan DM Capek KD , et al. Inhalation injury in the burned patient. Ann Plast Surg 2018; 80: S98–S105. 10.1097/SAP.0000000000001377 PMC5825291 29461292

[5] Wang B Chenru W Jiang Y , et al. Incidence and mortality of acute respiratory distress syndrome in patients with burns: a systematic review and meta-analysis. Front Med (Lausanne). Sec. Intensive Care Medicine and Anesthesiology 2021; 8. DOI: 10.3389/FMED.2021.709642/FULL. PMC8634659 34869410

[6] Field DJ Davis C Elbourne D , et al. UK Collaborative randomised trial of neonatal extracorporeal membrane oxygenation. Lancet 1996; 348: 75–82. 8676720

[7] Peek GJ Mugford M Tiruvoipati R , et al. Efficacy and economic assessment of conventional ventilatory support versus extracorporeal membrane oxygenation for severe adult respiratory failure (CESAR): a multicentre randomised controlled trial. Lancet 2009; 374: 1351–1363. 19762075 10.1016/S0140-6736(09)61069-2

[8] Ombrellaro M Goldthorn JF Harnar TJ , et al. Extracorporeal life support for the treatment of adult respiratory distress syndrome after burn injury. Surgery 1994; 115: 523–526. 8165546

[9] Patton ML Simone MR Kraut JD , et al. Successful utilization of ECMO to treat an adult burn patient with ARDS. Burns 1998; 24: 566–568. 9776097 10.1016/s0305-4179(98)00067-9

[10] Asmussen S Maybauer DM Fraser JF , et al. Extracorporeal membrane oxygenation in burn and smoke inhalation injury. Burns 2013; 39: 429–435. 23062623 10.1016/j.burns.2012.08.006

[11] Hsu PS Tsai YT Lin CY , et al. Benefit of extracorporeal membrane oxygenation in major burns after stun grenade explosion: Experience from a single military medical center. Burns 2017; 43: 674–680. 28040370 10.1016/j.burns.2016.08.035

[12] Szentgyorgyi L Shepherd C Dunn KW , et al. Extracorporeal membrane oxygenation in severe respiratory failure resulting from burns and smoke inhalation injury. Burns 2018; 44: 1091–1099. 29500117 10.1016/j.burns.2018.01.022

[13] Burke CR Chan T McMullan DM . c. Journal of Burn Care and Research 2017; 38: 174–178. 27606550 10.1097/BCR.0000000000000436

[14] Ainsworth CR Dellavolpe J Chung KK , et al. Revisiting extracorporeal membrane oxygenation for ARDS in burns: A case series and review of the literature. Burns 2018; 44: 1433–1438. 29903600 10.1016/j.burns.2018.05.008

[15] Kennedy JD Thayer W Beuno R , et al. ECMO in major burn patients: Feasibility and considerations when multiple modes of mechanical ventilation fail. Burns Trauma 2017; 5. DOI: 10.1186/s41038-017-0085-9. PMC5477428 28649575

[16] Nosanov LB McLawhorn MM Cruz MV , et al. A national perspective on ECMO utilization use in patients with burn injury. J Burn Care Res 2018; 39: 10–14. 10.1097/BCR.0000000000000555 28368919

[17] Makdisi G Wang IW . Extra corporeal membrane oxygenation (ECMO) review of a lifesaving technology. J Thorac Dis 2015; 7: E166–E176. 10.3978/j.issn.2072-1439.2015.07.17 PMC4522501 26380745

[18] Murphy DA Hockings LE Andrews RK , et al. Extracorporeal membrane oxygenation—hemostatic complications. Transfus Med Rev 2015; 29: 90–101. 25595476 10.1016/j.tmrv.2014.12.001

[19] Holzer F Gruber M Philipp A , etal. Predictors of bleeding in ECMO patients undergoing surgery. Minerva anestesiologica 2020; 86(1): 47–55. DOI: 10.23736/S0375-9393.19.13713-3. 31630509

[20] Olson SR Murphree CR Zonies D , etal. Thrombosis and Bleeding in Extracorporeal Membrane Oxygenation (ECMO) Without Anticoagulation: A Systematic Review. ASAIO journal (American Society for Artificial Internal Organs : 1992) 2021; 67(3): 290–296. 33627603 10.1097/MAT.0000000000001230 PMC8623470

[21] Eldredge RS Zhai Y Cochran A . Effectiveness of ECMO for burn-related acute respiratory distress syndrome. Burns : journal of the International Society for Burn Injuries 2019; 45(2): 317–321. DOI: 10.1016/j.burns.2018.10.012. 30429074

[22] Heng X Cai P Yuan Z , et al. Efficacy and safety of extracorporeal membrane oxygenation for burn patients: a comprehensive systematic review and meta-analysis. Burns Trauma 2023 Mar 1; 11: tkac056. 10.1093/burnst/tkac056 PMC9977350 36873286

[23] Schou A Mølgaard J Andersen LW , et al. Ethics in extracorporeal life support: a narrative review. Crit Care 2021 Jul 21; 25: 56. 34289885 10.1186/s13054-021-03689-0 PMC8293515

[24] Meltzer EC Ivascu NS Stark M , et al. A survey of Physicians’ attitudes toward decision-making authority for initiating and withdrawing VA-ECMO: results and ethical implications for shared decision making. J Clin Ethics 2016 Winter; 27: 281–289.. PMID: 28001135; PMCID: PMC5735424. 28001135 10.2217/bmm.10.117 PMC5735424

